# Nanoscopic morphological effect on the optical properties of polymer-grafted gold polyhedra†

**DOI:** 10.1039/d1na00035g

**Published:** 2021-02-10

**Authors:** Jaedeok Lee, Cheongwon Bae, Zihao Ou, Suhyeon Park, Jeongeon Kim, Juyeong Kim

**Affiliations:** Department of Chemistry and Research Institute of Natural Sciences, Gyeongsang National University Jinju 52828 South Korea chris@gnu.ac.kr; Department of Materials Science and Engineering, Stanford University Stanford California 94305 USA

## Abstract

Plasmonic nanoparticles show highly sensitive optical properties upon local dielectric environment changes. Hybridisation of plasmonic nanoparticles with active polymeric materials can allow stimuli-responsive and multiplex sensing over conventional monotonic sensing capacity. Such heterogeneous adlayers around the plasmonic core component, however, are likely to perturb the local refractive index in the nanometre regime and lead to uncertainty in its intrinsic sensitivity. Herein we prepare a series of polystyrene-grafted polyhedral gold nanoparticles, cubic and concave cubic cores, with different edge lengths and polymer thicknesses with precise synthesis control. Their localised surface plasmon resonance (LSPR) spectral changes are monitored to understand the effect of core morphological details in the interplay of nanoscale polymeric shells. Quantitative image analysis of changes in the core and shell shape contours and finite-difference time-domain simulations of the corresponding LSPR spectra and electric field distributions reveal that the magnitude of the LSPR spectral shift is closely dependent on the core morphology, polymer shell thickness and electric field intensity. We also demonstrate that the polystyrene-grafted gold concave cube displays higher sensitivity for nanoscale refractive index change in the polymer shell than the polystyrene-grafted gold cube at different temperatures. Our systematic investigation will help design polymer-composited plasmonic nanosensors for desirable applications.

## Abstract 2

We investigate a series of polymer-grafted polyhedral gold nanoparticles to find a correlation between localised surface plasmon resonance features and nanoscopic morphology details of the polyhedral core under the influence of the polymeric shell.

## Graphical abstract

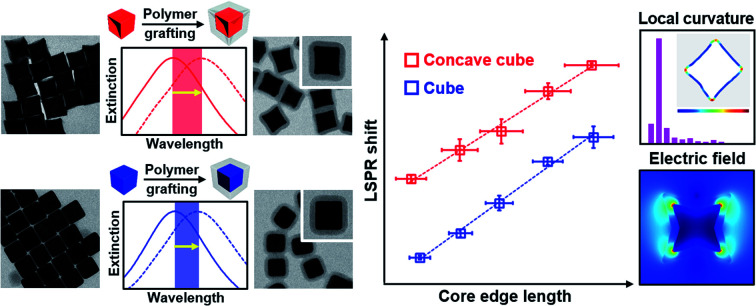

## Introduction

Plasmonic sensing has been developed using noble metal nanoparticles that possess localised surface plasmon resonance (LSPR).^1–3^ Spectral features corresponding to LSPR of the nanoparticle depend on its size, shape, elemental composition and surrounding dielectric environment.^4–8^ In particular, such spectral responsiveness to changes in the refractive index of the surrounding medium such as molecular adsorbates and solvent molecules allows the plasmonic nanoparticle as an excellent signal transducer in optical sensing.^9–12^ The LSPR peak of the plasmonic nanoparticle generally shifts to longer wavelengths as the refractive index of its surrounding environment increases, where the extent of the wavelength shift per unit refractive index determines detection sensitivity.^13,14^ Different dimensions and geometries of the plasmonic nanoparticle have been examined to achieve high refractive index sensitivity.^6,8,15^ Anisotropic nanostructures have shown enhanced sensitivity in comparison with spherical nanoparticles, and a gold nanorod with a high aspect ratio has resulted in higher refractive index sensitivity.^8^ In addition to modulation of the inherent properties of the plasmonic nanoparticle, its detection sensitivity could be improved by interfacial engineering with external entities such as polymeric layers and solid substrates.^16–18^ This approach can simultaneously promote monotonic sensing capacity to active and multiplex plasmonic sensing.^19^

Polymer-composited plasmonic nanoparticles have displayed versatile modes of plasmonic sensing due to reconfigurability and chemical responsiveness of the polymer layer,^20–23^ which would further facilitate mechanical flexibility and signal enhancement over conventional plasmonic sensing devices.^24^ For instance, gold nanoparticles coated with electroactive polymeric shells could give rise to active and reversible plasmonic switching performances at different electrochemical potentials.^25^ The pH-responsive polymer could induce plasmonic nanoparticles in the polymer matrix to aggregate and allow significant change in the LSPR peak through the plasmonic coupling effect.^26,27^ Although polymer-composited plasmonic nanoparticles have been developed *via* various approaches for desirable sensing functions with superior detectability, there has been lack of fundamental understanding on the correlation between the inherent properties of the plasmonic nanoparticle and its LSPR spectral features in the interplay with the polymer component. In addition to the bulk surrounding dielectric medium, such polymer layers would perturb the local refractive index in the nanometre regime around the plasmonic nanostructure, and it may cause unwanted disruption in the LSPR signal that can degrade its intrinsic sensing capacity. Thus, it is necessary to establish a systematic framework that elucidates the interaction between nanoscopic structural factors in the polymer-composited plasmonic nanoparticle and its LSPR band change, so that rational design of such hybrid plasmonic nanostructures can be realised for effective sensing material development.

We investigated a series of polymer-grafted polyhedral gold nanoparticles to find a correlation between LSPR features and nanoscopic morphology details of the polyhedral gold core under the influence of the polymeric shell layer. Two types of shape-anisotropic gold polyhedra, simple cubes and concave cubes, could be consistently prepared with different sizes. Spectral features of the polymer-grafted gold polyhedra were examined with respect to different edge lengths while maintaining the polymeric layer thickness constant. Also, their LSPR shifts upon nanoscale local refractive index modulation could be studied by applying the polymeric layer with different number-average molecular weights. Quantitative analysis of nanoscopic morphology details of the polymer-grafted gold polyhedra in relation with LSPR spectral shifts and finite-difference time-domain (FDTD) simulations were conducted to establish a systematic framework that could help to find proper design rules of polymer-composited plasmonic nanostructures.

## Results and discussion

**Fig. 1:**
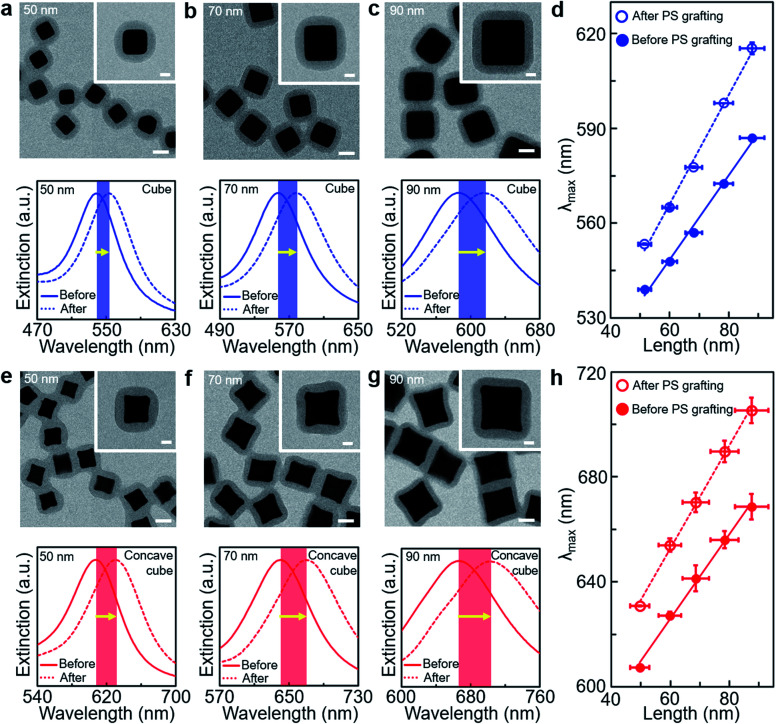
(a–c) Representative TEM images and normalised UV-visible spectra of PS-grafted gold cubes with different edge lengths (∼50 nm (a), ∼70 nm (b) and ∼90 nm (c)). The solid blue line in each spectral graph corresponds to gold cubes in water before the PS grafting, and the dotted blue line in each spectral graph corresponds to gold cubes in DMF after the PS grafting. (d) Plots of the LSPR band position as a function of the edge length of gold cubes before (filled blue circle) and after (unfilled blue circle) the PS grafting. (e–g) Representative TEM images and normalised UV-visible spectra of PS-grafted gold concave cubes with different edge lengths (∼50 nm (e), ∼70 nm (f) and ∼90 nm (g)). The solid red line in each spectral graph corresponds to gold concave cubes in water before the PS grafting, and the dotted red line in each spectral graph corresponds to gold concave cubes in DMF after the PS grafting. (h) Plots of the LSPR band position as a function of the edge length of gold concave cubes before (filled red circle) and after (unfilled red circle) the PS grafting. Scale bars: 50 nm for the large-scale images in (a–c) and (e–g) and 20 nm for the inset images in (a–c) and (e–g).

A series of gold nanocubes and concave nanocubes with similar edge lengths were synthesised *via* a seed-mediated growth method.^28^ The concave cube, in particular, has six {720} faceted concave planes with sharp edges and vertices,^29^ whereas the simple cube consists of six {100} faceted flat planes.^28^ The size and shape of both nanostructures were prepared highly uniformly by using uniform gold nanospheres that were obtained through iterative etching and regrowth of gold nanorods (Fig. S1†). We varied the amount of gold nanospheres to acquire different sizes of gold cubes and concave cubes (see the ESI† for details). The edge length of the cube could be controlled from 87.9 ± 4.1 nm to 51.5 ± 2.2 nm (Fig. S2, ESI†), and that of the concave cube could be controlled from 87.5 ± 5.7 nm to 49.6 ± 3.2 nm (Fig. S3, ESI†). Then, their surfaces were grafted with thiol-terminated polystyrene (PS) whose number-average molecular weight (*M*_n_) was 50 000 g mol^−1^. Note that the original nanoparticle surface ligand, cetyltrimethylammonium bromide (CTAB), was mostly removed through iterative centrifugations with deionised water for facile PS grafting. We optimised the PS concentration with respect to the nanoparticle size and shape, so that uniform PS grafting on the nanoparticle core could be obtained regardless of its dimension and geometry (see the ESI† for details). Transmission electron microscopy (TEM) images (Fig. 1 and S4, ESI†) showed that the PS shell was formed evenly along with the nanoparticle core boundary, where the PS shell with low atomic-weight elements was distinguished by lower contrast values than the gold nanoparticle core. The PS shell thickness was measured to be ∼20 nm for both core shapes even with different edge lengths (Table S1, ESI†), which allowed us to estimate the PS grafting density consistent over different core shapes and sizes.^30^ The PS shell layer adopted the core particle shape morphology, showing that the PS shell grafted on the concave cube core retained the morphology of concave edges and sharp tips.

The LSPR spectra of gold cubes and concave cubes before and after the PS grafting were investigated by UV-visible spectroscopy. As the edge length of the cube increased from ∼50 nm to ∼90 nm, its LSPR band position was measured with shifting from 539 nm to 587 nm before the PS grafting (Fig. 1a–d and Fig. S5, ESI†). After the PS grafting, the LSPR band position of the cube shifted to higher wavelengths than that without the PS shell. This spectral shift could be caused by local refractive index modulation around the cube core, which originated from the nanoscale PS shell layer formed on the particle surface. The refractive index of PS is reported to be 1.59, higher than that of *N*,*N*-dimethylformamide (DMF) used as solvent medium.^31^ The magnitude of such red-shift of the LSPR band by the PS shell layer would be smaller than in bulk PS medium, and there may be some effect by the solvent molecules in the PS shell matrix. We excluded such solvent effect in our spectral shift analysis, in that it would be insignificant for our comparative study with respect to the core size and geometry. In addition, the magnitude of such LSPR band shift in the PS-grafted cube was found to increase as the edge length of the cube core increased. The slope value derived from the LSPR band positions (Fig. 1d) was estimated to be 1.33 before the PS grafting and 1.73 after the PS grafting, implying that the cube with longer edge lengths has higher refractive index response to the PS shell with the same thickness. The dimensional increase range in the cube core was calculated to be larger than that in the PS shell dimension, based on the ratio of the PS shell volume to the cube core volume (Table S1, ESI†). We suppose that the dimension of the plasmonic nanoparticle could play a significant role in the inclined spectral shift under such nanoscale local refractive index modulation. The concave cube before the PS grafting also displayed the edge length-dependent increase in the LSPR band position, from 607 nm with ∼50 nm in the edge length to 669 nm with ∼90 nm in the edge length (Fig. 1e–h and S5, ESI†). Its LSPR band position was measured at a higher wavelength than that of the cube with a similar edge length. Such a difference could be attributed to its distinct morphology with sharp edges and vertices.^32^ Analogous to the increase of the LSPR spectral shift observed in the cube after the PS grafting, the LSPR band position of the PS-grafted concave cube shifted to higher wavelengths due to the local refractive index change by the PS shell.

**Fig. 2:**
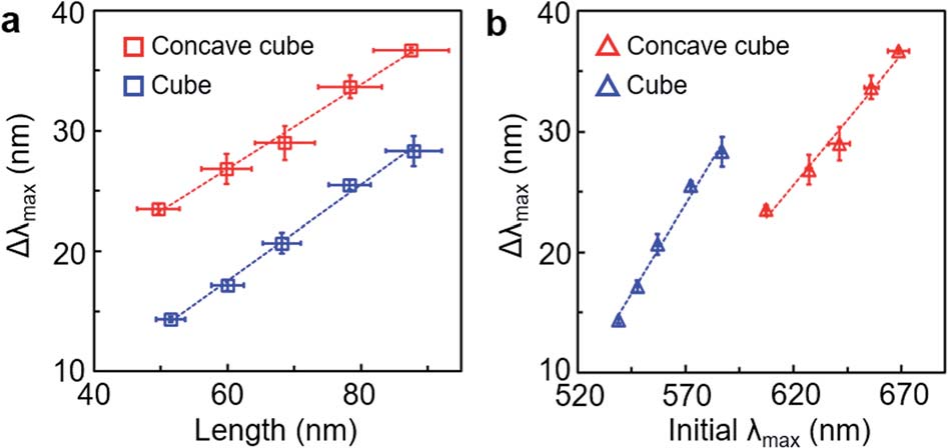
(a) Plots of LSPR spectral shift as a function of the edge length of PS-grafted gold cubes (blue unfilled square) and PS-grafted gold concave cubes (red unfilled square). (b) Plots of LSPR spectral shift as a function of the initial LSPR band position before the PS grafting (blue unfilled triangle: PS-grafted gold cubes, red unfilled triangle: PS-grafted gold concave cubes).

Core-geometric dependency for the LSPR spectral shift by the PS shell layer could be further analysed as a function of the core edge length and initial LSPR band position (Fig. 2). As the core edge length increased, the LSPR spectral shift by the PS shell increased from 14 nm to 28 nm for the cube and from 24 nm to 37 nm for the concave cube (Fig. 2a). At the same edge length, the extent of the LSPR spectral shift in the PS-grafted concave cube was measured to be larger by ∼10 nm than that of the PS-grafted cube. The LSPR spectral change is related to bulk refractive index sensitivity. The sensitivity for detecting refractive index changes depends on the magnitude of the spectral change, Δ*λ*_max_ = *η*_B_Δ*n*, where Δ*λ*_max_ is the LSPR spectral shift, *η*_B_ is the bulk refractive index sensitivity and Δ*n* is the refractive index change of the surrounding dielectric medium.^8^ Since the PS shell thickness could be maintained consistently in both nanostructures, an influence by local refractive index modulation, Δ*n*, would be almost identical in the two particles. We could presume that the difference in the core geometry had a significant impact on the nanoscopic refractive index sensitivity, Δ*λ*_max_, allowing the concave cube core to have a higher *η*_B_ value than the cube core at the same edge length. It could also be demonstrated by higher initial LSPR band positions in the PS-grafted concave cube than those in the PS-grafted cube when considering each core shape with the same edge length (Fig. 2b). The concave geometry in the PS-grafted gold polyhedra would be beneficial to achieve efficient refractive index sensitivity with the same quantity of material consumption.

**Fig. 3:**
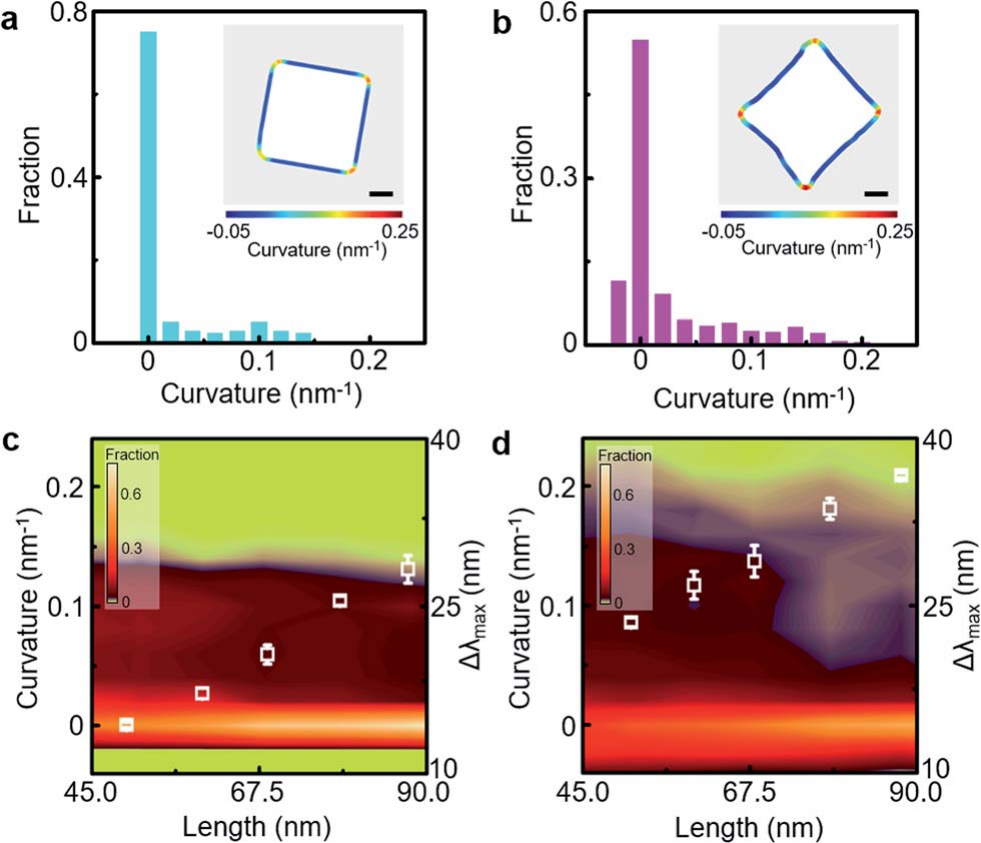
(a and b) Representative curvature distribution of the core contour in the PS-grafted gold cube (a) and PS-grafted gold concave cube (b) with ∼90 nm edge length (inset: overlaid surface contour colour-coded according to local surface curvature values of the core from a TEM image). (c and d) Correlation map of curvature distribution as a function of the edge length of PS-grafted gold cubes (c) and PS-grafted gold concave cubes (d). The corresponding LSPR spectral shift values (white unfilled square) are overlaid on the correlation map. Scale bar: 20 nm.

We quantitatively investigated the correlation between the core geometry and LSPR spectral shift through TEM image analysis of the PS-grafted gold polyhedra (Fig. 3). The local curvature value of the core particle contour as a representative indicator of the core geometry was quantified *via* an open-source software ImageJ and our customised MATLAB code (Fig. S6, ESI†).^33^ The core contour could be colour-coded according to the extent of the local curvature value, the inverse of the radius of the best-fitted circle. High curvature values emerged mostly around core vertices in the PS-grafted gold polyhedra (Fig. S7, ESI†). The curvature distribution for the core contour in the PS-grafted cube with ∼90 nm edge length contained a dominant fraction near 0 nm^−1^ curvature (Fig. 3a), reflecting that the majority of the core contour was occupied by flat edges. As the edge length of the PS-grafted cube decreased, the fraction near 0 nm^−1^ curvature decreased down to 0.44 (Fig. S7, ESI†). On the other hand, the curvature distribution for the core contour in the PS-grafted concave cube with ∼90 nm edge length spreads between −0.05 nm^−1^ and 0.2 nm^−1^ (Fig. 3b), where the portion of negative curvature values corresponded to the concave surface contour than the flat edge. The concave cube core contained extended fractions of higher curvature values in comparison with the cube core, which resulted from sharp vertices derived from intersections of concave edges. The correlation map for curvature distribution over different edge lengths on each core shape clearly demonstrated close core-geometric dependency on the LSPR spectral shift (Fig. 3c and d). Curvature distribution in the PS-grafted cube with different edge lengths indicated an increase in the portion of low curvature value near 0 nm^−1^ as the edge length increased, and the portion of high curvature values over 0.15 nm^−1^ was hardly seen. The PS-grafted concave cube exhibited a wide portion of high curvature values over 0.15 nm^−1^ along with the whole edge length range in contrast to the PS-grafted cube. Thus, we could conclude that nanoscopic morphological details with high curvature values were responsible for the larger LSPR shift in the PS-grafted gold polyhedra.

**Fig. 4:**
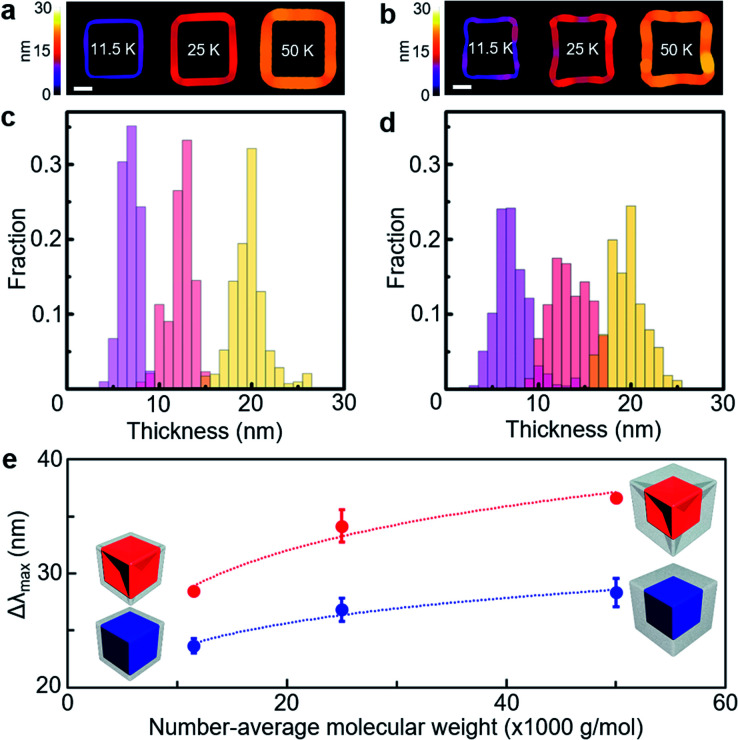
(a and b) Singled-out PS shell area on the core colour-coded according to the local thickness values in the PS-grafted gold cube (a) and PS-grafted gold concave cube (b) with different number-average molecular weights of PS (*M*_n_ = 11 500 g mol^−1^ for 11.5 K, 25 000 g mol^−1^ for 25 K and 50 000 g mol^−1^ for 50 K). (c and d) Local thickness distribution of the PS shell in the PS-grafted gold cube (c) and PS-grafted gold concave cube (d) with different number-average molecular weights of PS (*M*_n_ = 11 500 g mol^−1^ for light purple bars, 25 000 g mol^−1^ for light red bars and 50 000 g mol^−1^ for light yellow bars). (e) Plot of LSPR spectral shift as a function of the number-average molecular weight of PS (blue filled circle: PS-grafted gold cubes, red filled circle: PS-grafted gold concave cubes). The dotted lines are a guide to the eye. The core particle is presented as red for the concave cube and blue for the cube in the schematics, and the PS shell is presented as grey. Scale bar: 30 nm.

The core morphology-dependent modulation in the LSPR spectral shift could be further examined as a function of the local PS shell thickness (Fig. 4). We applied different number-average molecular weights of PS (*M*_n_ = 11 500 g mol^−1^, 25 000 g mol^−1^ and 50 000 g mol^−1^) on each core shape with ∼90 nm edge length, respectively. The local PS shell thickness in the PS-grafted gold polyhedra could be quantified with the two-dimensional projected shape of the PS shell in the TEM image (Fig. 4a and b and Fig. S8, ESI†). For the PS-grafted cube (Fig. 4c), the distribution of the local PS shell thickness showed the highest fraction at ∼8 nm for PS (*M*_n_ = 11 500 g mol^−1^), ∼13 nm for PS (*M*_n_ = 25 000 g mol^−1^) and ∼20 nm for PS (*M*_n_ = 50 000 g mol^−1^), which confirmed that the local PS shell thickness was proportional to its number-average molecular weight. The distribution of the local PS shell thickness spreads out as the number-average molecular weight of PS increased, which could be caused by a relative increase of the corner area. For the PS-grafted concave cube (Fig. 4d), the distribution of the local PS shell thickness showed the highest fraction at ∼7 nm for PS (*M*_n_ = 11 500 g mol^−1^), ∼12 nm for PS (*M*_n_ = 25 000 g mol^−1^) and ∼20 nm for PS (*M*_n_ = 50 000 g mol^−1^). The distribution of the local PS shell thickness in the PS-grafted concave cube was more spread in comparison with that in the PS-grafted cube. Such extended distribution could be ascribed to a higher portion of uneven core surface contour in the PS-grafted concave cube owing to its concave edges and sharp vertices. The LSPR spectra of the PS-grafted gold polyhedra were measured to find the effect of the PS shell thickness change (Fig. 4e). The LSPR spectral shift ranged from 24 nm for PS (*M*_n_ = 11 500 g mol^−1^) to 28 nm for PS (*M*_n_ = 50 000 g mol^−1^) in the PS-grafted cube, and from 29 nm for PS (*M*_n_ = 11 500 g mol^−1^) to 37 nm for PS (*M*_n_ = 50 000 g mol^−1^) in the PS-grafted concave cube. Such PS shell thickness dependency on the LSPR spectral shift could be accounted for by a modified expression of the LSPR spectral shift, Δ*λ*_max_ = *η*_B_(*n*_analyte_ − *n*_medium_) (1 − e^(−2*d*/*L*)^), where *n*_analyte_ is the refractive index of the analyte, *n*_medium_ is the refractive index of the surrounding medium, *d* is the thickness of the adsorbed layer and *L* is the decay length of the electric field.^8^ As the increase of the PS shell thickness corresponded to the *d* value, the above equation could support our observation of the increase in the LSPR spectral shift as a function of the PS shell thickness. In addition, the PS-grafted concave cube showed larger LSPR spectral shifts than the PS-grafted cube over the examined range of PS shell thicknesses, which confirms that the concave cube core has superior refractive index sensitivity upon a few nanometre changes in the surrounding polymer thickness.

**Fig. 5:**
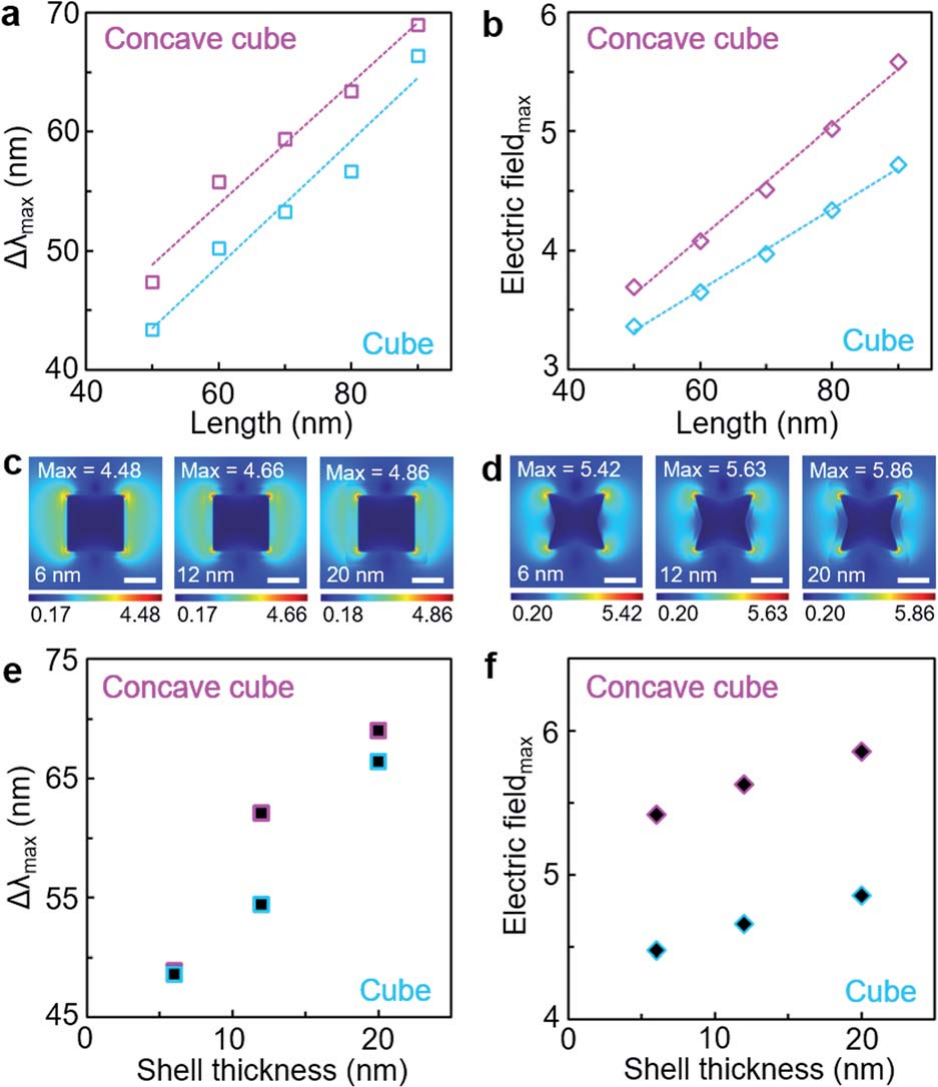
FDTD simulations of PS-grafted gold cubes and concave cubes. (a) Plot of simulated LSPR spectral shift as a function of the edge length of PS-grafted gold cubes (sky blue unfilled square) and PS-grafted gold concave cubes (magenta unfilled square). (b) Plot of maximum electric field intensity as a function of the edge length of PS-grafted gold cubes (sky blue unfilled rhombus) and PS-grafted gold concave cubes (magenta unfilled rhombus). (c and d) Electric field distribution map of PS-grafted gold cubes (c) and PS-grafted gold concave cubes (d) with different polymer shell thicknesses (6 nm, 12 nm and 20 nm, from left to right). (e) Plot of simulated LSPR spectral shift as a function of the PS shell thickness of PS-grafted gold cubes (sky blue filled square) and PS-grafted gold concave cubes (magenta filled square). (f) Plot of maximum electric field intensity as a function of the PS shell thickness of PS-grafted gold cubes (sky blue filled rhombus) and PS-grafted gold concave cubes (magenta filled rhombus). Scale bar: 50 nm.

We implemented FDTD simulations for LSPR spectra and electric field distributions of the PS-grafted gold polyhedra to seek a correlation between LSPR spectral shift and electric field enhancement (Fig. 5). The LSPR spectral shift derived from the simulated spectra of the PS-grafted gold polyhedra with different edge lengths showed an increasing trend with longer edge lengths in both core shapes and a larger variation in the concave cube core than in the cube core with the same edge length (Fig. 5a, S9 and S10, ESI†), which corresponded to our experimental spectral behaviour. Note that there are consistent deviations in the LSPR peak positions between the measured and simulated spectra, which could be ascribed to inhomogeneous core morphology and potential interaction between the polymer shell matrix and solvent molecules. The linear relationship between the core edge length and LSPR spectral shift could be attributed to the electromagnetic retardation affected by the nanoparticle dimension.^5,34^ Maximum electric field intensity values were estimated from electric field distribution maps of those PS-grafted gold polyhedra with different edge lengths (Fig. 5b, S11 and S12, ESI†). On this basis, we could confirm that there was close dependency of the LSPR spectral shift on the electric field enhancement, which originated from the core dimension and geometry. In addition, LSPR spectra and electric field distribution were simulated in the PS-grafted gold polyhedra with different PS shell thicknesses, 6 nm, 12 nm and 20 nm (Fig. 5c–f and S13, ESI†). As the PS shell thickness increased, the corresponding LSPR spectral shift became larger as observed experimentally (Fig. 5e). With the same PS shell thickness, the concave cube core displayed a larger LSPR spectral shift than the cube core. Over the whole range of the PS shell thicknesses, maximum electric field intensity values were estimated higher for the concave cube core than the cube core (Fig. 5f). The electric field enhancement factor could have a significant impact on the magnitude of the LSPR spectral shift, resulting in higher refractive index sensitivity by the plasmonic core morphology with higher curvature values.

**Fig. 6:**
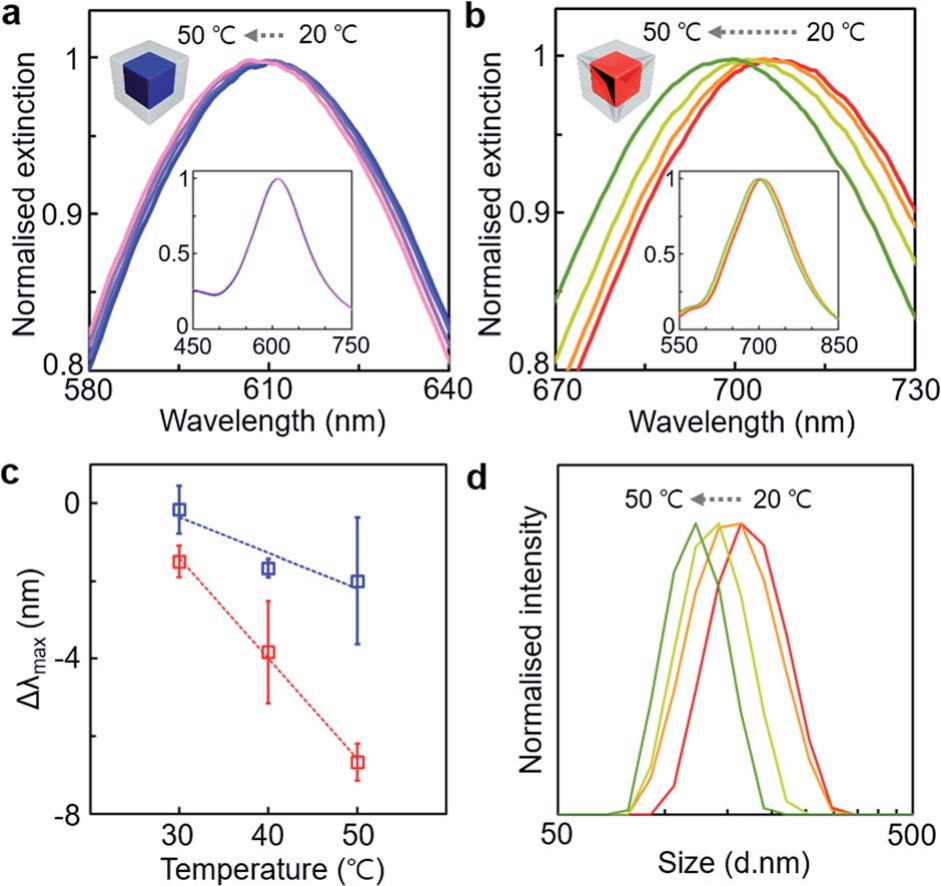
(a and b) Normalised UV-visible spectra of the PS-grafted gold cube (a) and PS-grafted gold concave cube (b) from 20 °C to 50 °C (inset: the corresponding spectra before magnification). (c) Plot of LSPR spectral shift as a function of temperature of the PS-grafted gold cube (blue unfilled square) and PS-grafted gold concave cube (red unfilled square). (d) Normalised DLS spectra of the PS-grafted gold concave cube from 20 °C to 50 °C.

The superior sensitivity of the PS-grafted gold concave cube on nanoscale local refractive index change could be further demonstrated at different temperatures (Fig. 6). The LSPR spectra of the PS-grafted gold cube and concave cube with ∼90 nm edge length and ∼20 nm PS shell thickness were monitored respectively upon heating their colloidal solutions from 20 °C to 50 °C. The PS-grafted cube showed a minimal blue-shift of the LSPR band position by 2 nm from 613 nm to 611 nm (Fig. 6a), whereas the blue-shift of the LSPR band in the PS-grafted concave cube was more pronounced by 7 nm from 707 nm to 700 nm (Fig. 6b). The magnitude of the LSPR spectral shift was gradually deviated as the temperature rose (Fig. 6c), confirming that the PS-grafted concave cube could express enhanced spectral responses to changes in the temperature. It appears that such a blue-shift in the LSPR spectra could be attributed to the local refractive index change due to partial detachment of the PS shell^35^ and decrease in the refractive index of the surrounding medium at the elevated temperatures,^36^ which could be supported with dynamic light scattering (DLS) spectra (Fig. 6d) as well as our observation in irreversible LSPR spectral shift without the core morphology being changed. Also, the partial release of the PS shell could be noticed by a slight increase of PS residues on the TEM grid of the samples treated at higher temperatures (Fig. S14, ESI†).

## Conclusions

We implemented a systematic study on the LSPR spectral shift of a series of polyhedral gold nanoparticles, simple cubes and concave cubes, whose surfaces were grafted with nanoscale polymer layers. The LSPR spectral shift in the PS-grafted gold polyhedra was measured to be proportional to the core edge length. In particular, the PS-grafted concave cube showed higher LSPR spectral shifts than the PS-grafted cube with similar edge lengths. Our quantitative TEM image analysis of the core curvature contour revealed a high correlation between the LSPR spectral shift and core geometry. The concave cube core contained extended fractions of high curvature values due to its concave edges and sharp vertices compared to the cube core, giving rise to larger LSPR spectral shifts in the presence of the polymer shell. The PS shell thickness could also be controlled in the PS-grafted gold polyhedra, provided that the concave cube core had higher sensitivity to nanoscale local refractive index change by the PS shell thickness modulation. Our experimental observation in the LSPR spectral changes could be demonstrated by FDTD simulations. The electric field distribution map showed higher electric field enhancement in the PS-grafted gold polyhedra with longer edge lengths, and the concave cube core retained higher maximum electric field intensity values than the cube core with the same edge length. Our understanding of the nanoscopic morphological effect on the LSPR spectra of PS-grafted gold polyhedra can be utilised for the development of polymer-composited plasmonic materials with enhanced and multiplex plasmonic sensing applications.

## Conflicts of interest

There are no conflicts to declare.

## Supplementary Material

NA-003-D1NA00035G-s001
