# Supplementary material for: Nanoscopic morphological effect on the optical properties of polymer-grafted gold polyhedra
Source: Nanoscale Adv. 2021 Feb 10;3(7):1927–33. doi: 10.1039/d1na00035g (PMC9419197; doi:10.1039/d1na00035g)
Supplement: NA-003-D1NA00035G-s001 [file NA-003-D1NA00035G-s001.pdf]

Electronic Supplementary Material (ESI) for Nanoscale Advances.
This journal is © The Royal Society of Chemistry 2021

Supplementary Information

Nanoscopic morphological effect on optical properties of polymer-grafted gold polyhedra

Jaedeok Lee,‡[a] Cheongwon Bae,‡[a] Zihao Ou,[b] Suhyeon Park,[a] Jeongeon Kim[a] and Juyeong Kim*[a]

[a]Department of Chemistry and Research Institute of Natural Sciences, Gyeongsang National University, Jinju 52828, South Korea

[b]Department of Materials Science and Engineering, Stanford University, Stanford, California 94305, United States

*To whom correspondence should be addressed. Email: chris@gnu.ac.kr

‡These authors contributed equally to this work.

Materials and Methods

Figures S1-S14

Table S1

References

Materials and Methods

Section 1. Chemicals

Sodium borohydride (99%, $NaBH_4$, Sigma-Aldrich), L-ascorbic acid (BioXtra, 99%, $C_6H_8O_6$, Sigma-Aldrich), gold(III) chloride trihydrate (99.9%, $HAuCl_4 \cdot 3H_2O$, Sigma-Aldrich), silver nitrate (99.0%, $AgNO_3$, Sigma-Aldrich), cetyltrimethylammonium bromide (99.9%, $C_{19}H_{42}BrN$, Sigma-Aldrich), cetylpyridinium chloride monohydrate (98%, $C_{21}H_{38}ClN \cdot H_2O$, TCI), potassium bromide (99%, KBr, Sigma-Aldrich), Hydrochloric acid (99.999%, HCl, Alfa Aesar), N,N-dimethylformamide (99.5%, C_3H_7NO, Samchun), and ω-thiol-terminated poly(styrene) (M_n = 11500 g/mol, 25000 g/mol and 50000 g/mol, Polymer Source) were purchased and used without further purification. All glassware for gold nanoparticle syntheses was treated with aqua regia (a mixture of HCl and HNO_3), thoroughly rinsed with deionized water and dried immediately before use. Deionized water (18.2 MΩ·cm at 25 ℃) purified by a Merck Millipore Direct Q3 UV Water Purification System was used for all solution preparation and washing.

Section 2. Synthesis of gold nanoparticles

2.1. Nanorods

Gold nanorods were synthesized by a seed-mediated growth method.[1,2] An aqueous solution of 10 mM $HAuCl_4$ (125 μL) was added to 5 mL of 100 mM cetyltrimethylammonium bromide (CTAB) in a 20 mL scintillation vial. While the mixture was stirred at 1150 rpm, an aqueous solution of $NaBH_4$ (10 mM, 300 μL) cooled in an ice bath was quickly injected to the solution. The gold seed solution was stirred for 1 min and left undisturbed at 30 °C for 20 min. Then, aqueous solutions of $HAuCl_4$ (10 mM, 10 mL), $AgNO_3$ (10 mM, 1.8 mL) and ascorbic acid (100 mM, 1.14 mL) were added in sequence into 200 mL of 100 mM CTAB in a 250 mL Erlenmeyer flask at 30 °C. The prepared seed solution (240 μL) was quickly injected to the mixture while stirred at 500 rpm, and left undisturbed at 30 °C for 2 h. Gold nanorods were collected after twice centrifugations (8000 rpm, 15 min) and dispersed in 5 mL of 50 mM CTAB (λ_{max} = 756 nm).

2.2. Nanospheres

Uniform gold nanospheres were prepared according to literature method with modification.[1,2] Test etching reactions were conducted to determine an appropriate volume of 10 mM $HAuCl_4$ that would be required for

spherical shape. Different volumes of 10 mM $HAuCl_4$ (3 µL, 3.5 µL, 4 µL, 4.5 µL, 5 µL, 5.5 µL and 6 µL) were added to 500 µL of the gold nanorod solution (extinction = 2 at 756 nm) in a 2 mL microtube, respectively, and placed in a thermomixer at 40 ℃ under 300 rpm for 4 h. After etching, their UV-visible spectra were measured, and an optimum volume of 10 mM $HAuCl_4$ (4.1 µL) could be determined. A scale-up etching reaction was conducted as follows. The gold nanorod solution (4.6 mL) was mixed with 50 mM CTAB (83.57 mL) in a 250 mL Erlenmeyer flask at 40 ℃. An aqueous solution of 10 mM $HAuCl_4$ (718 µL) was added to the mixture, and it was stirred at 200 rpm for 4 h. Gold nanospheres were collected after twice centrifugations (11000 rpm, 45 min) and dispersed in 100 mM cetylpyridinium chloride monohydrate (CPC).

In order to improve shape uniformity, regrowth and etching processes were applied to the prepared gold nanosphere. For regrowth, aqueous solutions of $HAuCl_4$ (10 mM, 642 µL), ascorbic acid (100 mM, 8.25 mL) and the nanosphere solution (11 mL) were added in sequence into 36.67 mL of 10 mM CPC in a 100 mL Erlenmeyer flask while stirred at 40 ℃ under 300 rpm. The mixture was left undisturbed for 15 min. The regrown gold nanospheres were collected through twice centrifugations (10000 rpm, 10 min) and dispersed in 50 mM CTAB. Then, etching of the regrown gold nanospheres was conducted by reacting the regrown nanoparticle solution (40 mL) with an aqueous solution of $HAuCl_4$ (10 mM, 240 µL) at 40 ℃ under 200 rpm for 4 h. Uniform gold nanospheres were collected through twice centrifugations (11000 rpm, 45 min) and dispersed in 100 mM CPC. In order to remove relatively large gold nanospheres, the product solution was centrifugated at 4000 rpm for 4 min and its supernatant was collected. The centrifugation was repeated four times to achieve better shape uniformity for the gold nanosphere.

2.3. Nanocubes

Gold nanocubes were prepared by a seed-mediated growth method.[1,2] Aqueous solutions of KBr (100 mM, 500 µL), $HAuCl_4$ (10 mM, 100 µL), ascorbic acid (100 mM, 150 µL) and the gold nanosphere solution (extinction = ~1 at 524 nm) were added in sequence into 5mL of 100 mM CPC in a 20 mL scintillation vial while mixed well at each addition. The mixture was left undisturbed for 1 h. Gold nanocubes were collected after twice centrifugations (4000 rpm, 10 min) and dispersed in 50 mM CTAB.

2.4. Concave nanocubes

Gold concave nanocubes were prepared by a seed-mediated growth method.[1,2] Aqueous solutions of HCl (1 M, 250 μL), $HAuCl_4$ (10 mM, 250 μL), $AgNO_3$ (10 mM, 62.5 μL), ascorbic acid (100 mM, 47.5 μL) and the gold nanosphere solution (extinction = ~1 at 524 nm) were added in sequence into 5 mL of 100 mM CPC in a 20 mL scintillation vial while mixed well at each addition. The mixture was left undisturbed for 2 h. Gold concave nanocubes were collected after twice centrifugations (2700 rpm, 7 min) and dispersed in 50 mM CTAB.

Section 3. Polymer grafting

Polymer grafting on the polyhedral gold nanoparticles was performed according to a modified literature method.[2,3] First, the CTAB concentration in the gold nanoparticle solution prepared from Section 2.4 and 2.5 was reduced through centrifugation and redispersion in deionized water. Then, the gold nanoparticle solution was concentrated by removing supernatant as much as possible, leaving ~10 μL of the nanoparticle solution. The concentrated particle solution was mixed well with 40 μL of deionized water, which was quickly injected into 1.07 mL of the polystyrene (PS) solution (0.1 mg/mL in N,N-dimethylformamide (DMF)) in a 5 mL scintillation vial while sonicated. The mixture was sonicated for 1 min and left undisturbed for 30 min. The PS-grafted polyhedral gold nanoparticles were collected after six-time centrifugations and dispersed in DMF.

Section 4. Characterisation

A FEI Tecnai 12 transmission electron microscope with a LaB_6 emitter at 120 kV was used for the nanoparticle core and polymer shell characterisation. Specimen was prepared by dropping 10 μL of CTAB-deficient particle solution on a TEM grid, which was fully dried at room temperature for 3 h before the imaging. For PS-grafted nanoparticles, the particle solution in DMF (10 μL) was dropped on a TEM grid, and the droplet was gently wiped after 30 min. UV-visible spectra were obtained using a Genesys 10S UV-Vis spectrophotometer with a quartz cuvette (path length = 1 cm). Temperature-dependent UV-visible spectra were measured using a Thermo Scientific Evolution 600. At each temperature, sample solution was stabilized for 15 min before spectral acquisition. Dynamic light scattering was measured by a Malvern Zetasizer Nano ZS.

Section 5. Image analysis

Curvature analysis was conducted by ImageJ and our customized Matlab code.[2] A TEM image of a PS-grafted gold nanoparticle was treated via ImageJ. First, the image type was changed to 8 bit, to which Gaussian blur was applied by sigma value of 2.0 pixels. The boundary of the core particle was cut clearly through polygon selections. Then, a core mask image was obtained using a threshold function. The mask image was further processed via our customized Matlab code, giving rise to curvature distribution and colour map.

Local thickness analysis was conducted by ImageJ.[2] A shell mask could be acquired by the same procedure used for the core mask. The boundaries of the core and shell mask were matched and overlapped each other, followed by applying threshold function and flood fill. Then, local thickness function was executed at the core-shell mask, giving rise to local thickness colour map and distribution.

Section 6. Finite-difference time-domain (FDTD) simulations

Three-dimensional (3D) FDTD simulations were preformed using the commercially available software package FDTD solutions (Lumerical 2020a R7). All 3D models were constructed and exported by 3D MAX software. The simulation boundary was set to perfectly matched layer. The dielectric constant for gold was taken from experimental bulk results previously reported.[4] The refractive index of the surrounding medium was set as 1.3330 for water, 1.4305 for DMF and 1.5916 for PS according to literature.[5] The total-field scattered-field function was used to obtain absorption and scattering spectra. The wavelength of source was adjusted from 300 nm to 1000 nm, and it was directed toward XY plane. The optimum override mesh (1 nm × 1 nm × 1 nm), mesh accuracy (level 6) and simulation size (3.5 μm) were set after convergence test for reliable data acquisition.

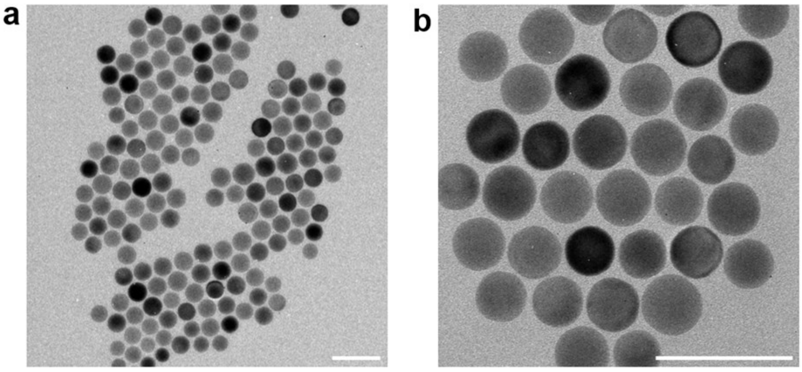

Fig. S1 (a) and (b) TEM images of gold nanospheres. Scale bars: 50 nm.

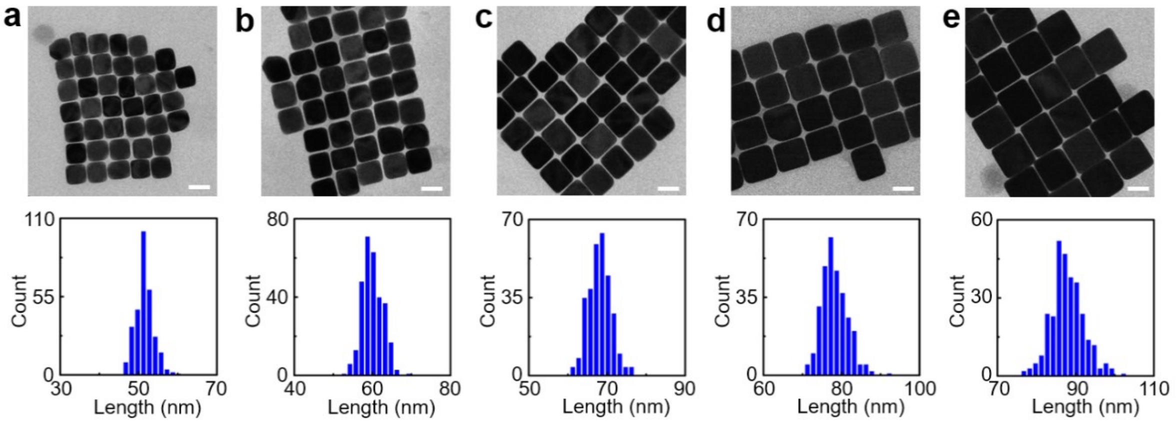

Fig. S2 Representative TEM images and length distribution histograms for gold nanocubes with edge length of (a) 51.5 ± 2.2 nm, (b) 60.0 ± 2.4 nm, (c) 68.1 ± 2.8 nm, (d) 78.3 ± 3.1 nm and (e) 87.9 ± 4.1 nm. Data were obtained by measuring 300 particles in each edge length. Scale bars: 50 nm.

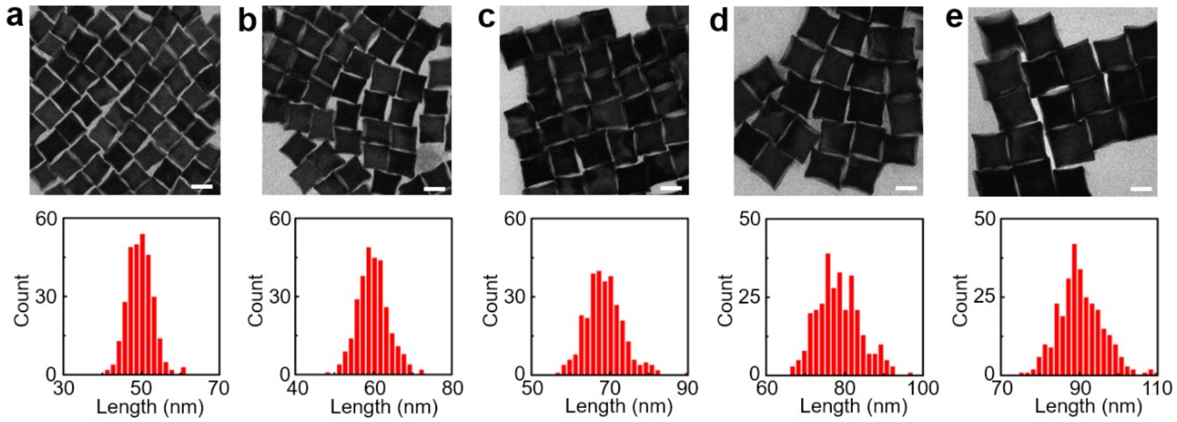

Fig. S3 Representative TEM images and length distribution histograms for gold concave nanocubes with edge length of (a) 49.6 ± 3.2 nm, (b) 59.8 ± 3.7 nm, (c) 68.6 ± 4.5 nm, (d) 78.3 ± 4.8 nm and (e) 87.5 ± 5.7 nm. Data were obtained by measuring 300 particles in each edge length. Scale bars: 50 nm.

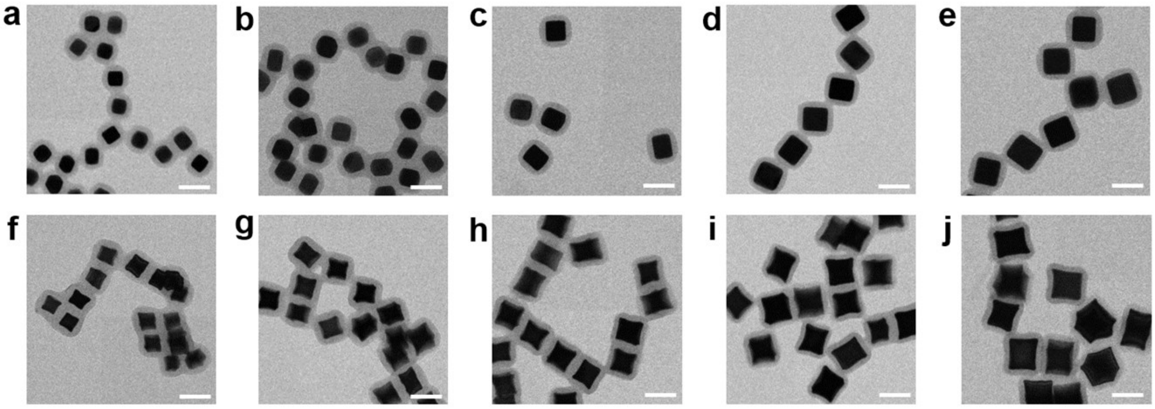

Fig. S4 Representative TEM images of PS-grafted gold nanocubes with edge length of (a) 51.5 ± 2.2 nm, (b) 60.0 ± 2.4 nm, (c) 68.1 ± 2.8 nm, (d) 78.3 ± 3.1 nm and (e) 87.9 ± 4.1 nm, and PS-grafted gold concave nanocubes with edge length of (f) 49.6 ± 3.2 nm, (g) 59.8 ± 3.7 nm, (h) 68.6 ± 4.5 nm, (i) 78.3 ± 4.8 nm and (j) 87.5 ± 5.7 nm. Scale bars: 100 nm.

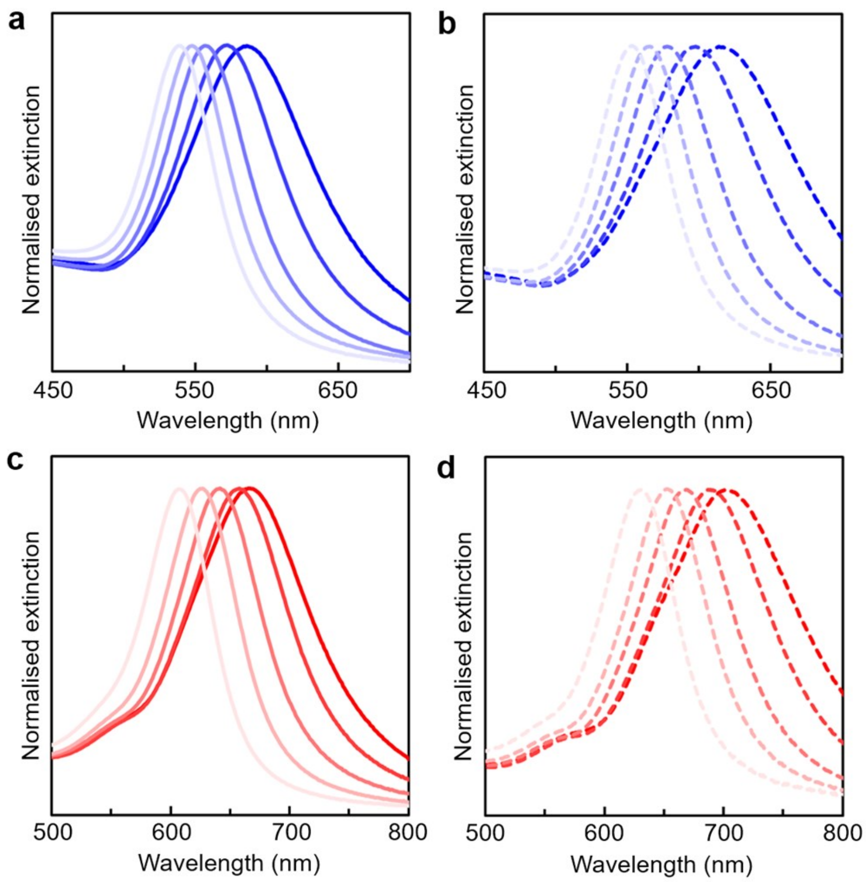

Fig. S5 (a) Representative normalised UV-visible spectra of gold nanocubes with different edge lengths (from left to right: ~50 nm, ~60 nm, ~70 nm, ~80 nm and ~90 nm in edge length). (b) Representative normalised UV-visible spectra of PS-grafted gold nanocubes with different edge lengths (from left to right: ~50 nm, ~60 nm, ~70 nm, ~80 nm and ~90 nm in edge length). (c) Representative normalised UV-visible spectra of gold concave nanocubes with different edge lengths (from left to right: ~50 nm, ~60 nm, ~70 nm, ~80 nm and ~90 nm in edge length). (d) Representative normalised UV-visible spectra of PS-grafted gold concave nanocubes with different edge lengths (from left to right: ~50 nm, ~60 nm, ~70 nm, ~80 nm and ~90 nm in edge length). The spectra of gold polyhedra before the PS grafting were measured in water, and those of PS-grafted gold polyhedra were measured in DMF.

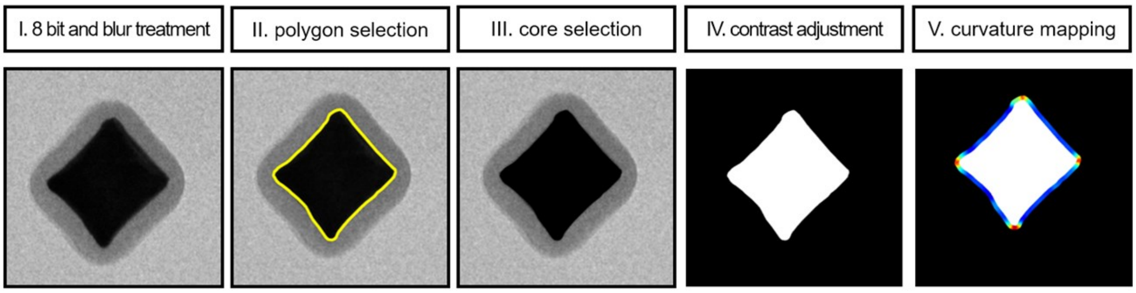

Fig. S6 Image analysis protocol for curvature mapping. Steps I-IV were conducted via ImageJ, and Step V was conducted via our customized Matlab code.

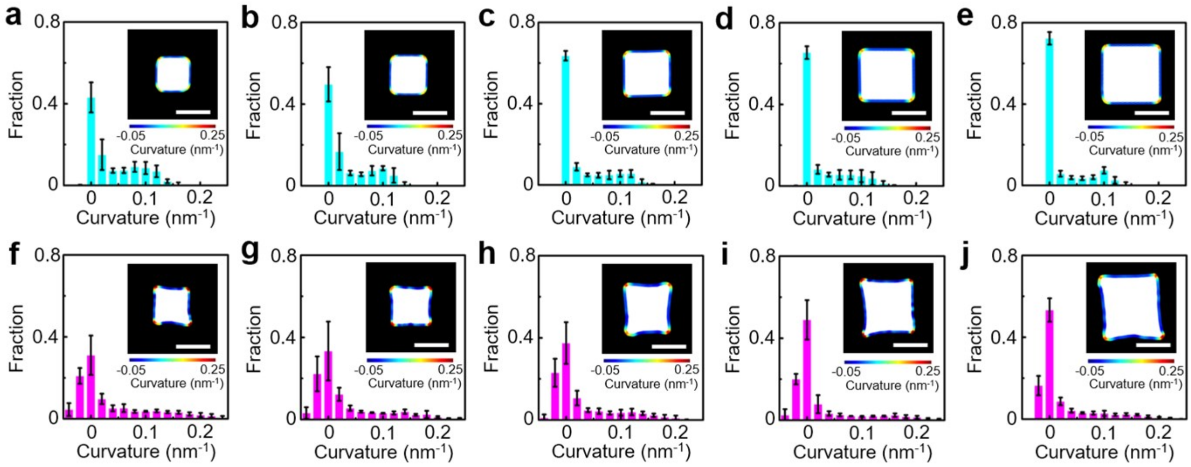

Fig. S7 (a-e) Curvature distribution of the cube core contour in the PS-grafted gold nanocube with different edge lengths ((a): ~50 nm, (b): ~60 nm, (c): ~70 nm, (d): ~80 nm and (e): ~90 nm in edge length). (f-j) Curvature distribution of the concave cube core contour in the PS-grafted gold concave nanocube with different edge lengths ((f): ~50 nm, (g): ~60 nm, (h): ~70 nm, (i): ~80 nm and (j): ~90 nm in edge length). Data were collected from 5 different particles in each histogram. The inset image shows a representative overlaid surface contour colour-coded according to local surface curvature values of the core from a TEM image. Scale bars: 50 nm.

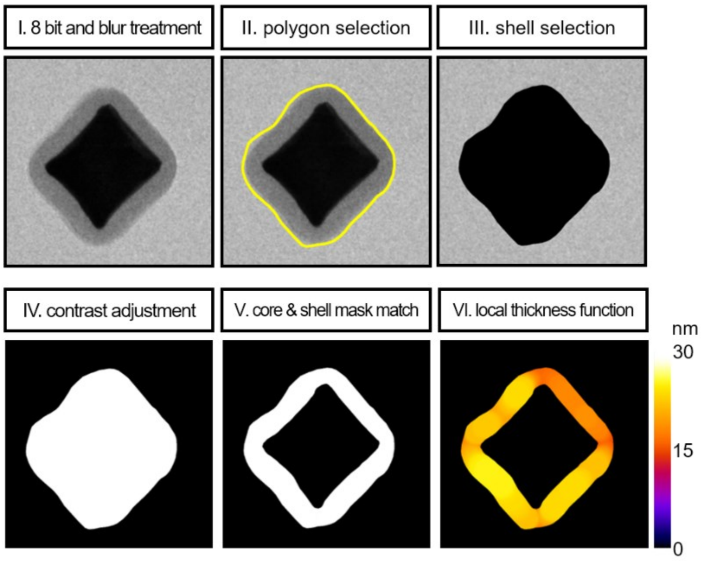

Fig. S8 Image analysis protocol for local thickness mapping. Steps I-VI were conducted via ImageJ.

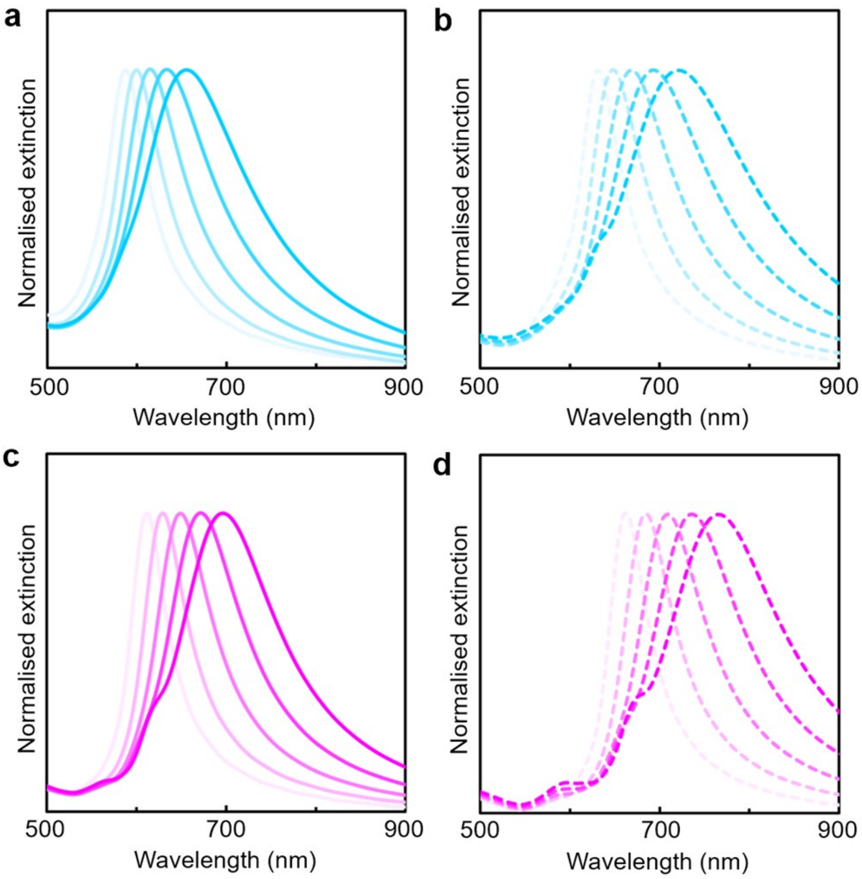

Fig. S9 (a) Simulated UV-visible spectra of gold nanocubes with different edge lengths (from left to right: 50 nm, 60 nm, 70 nm, 80 nm and 90 nm in edge length). (b) Simulated UV-visible spectra of PS-grafted gold nanocubes with different edge lengths (from left to right: 50 nm, 60 nm, 70 nm, 80 nm and 90 nm in edge length). (c) Simulated UV-visible spectra of gold concave nanocubes with different edge lengths (from left to right: 50 nm, 60 nm, 70 nm, 80 nm and 90 nm in edge length). (d) Simulated UV-visible spectra of PS-grafted gold concave nanocubes with different edge lengths (from left to right: 50 nm, 60 nm, 70 nm, 80 nm and 90 nm in edge length). The spectra of gold polyhedra before the PS grafting were simulated in water, and those of PS-grafted gold polyhedra (PS shell thickness = 20 nm) were simulated in DMF.

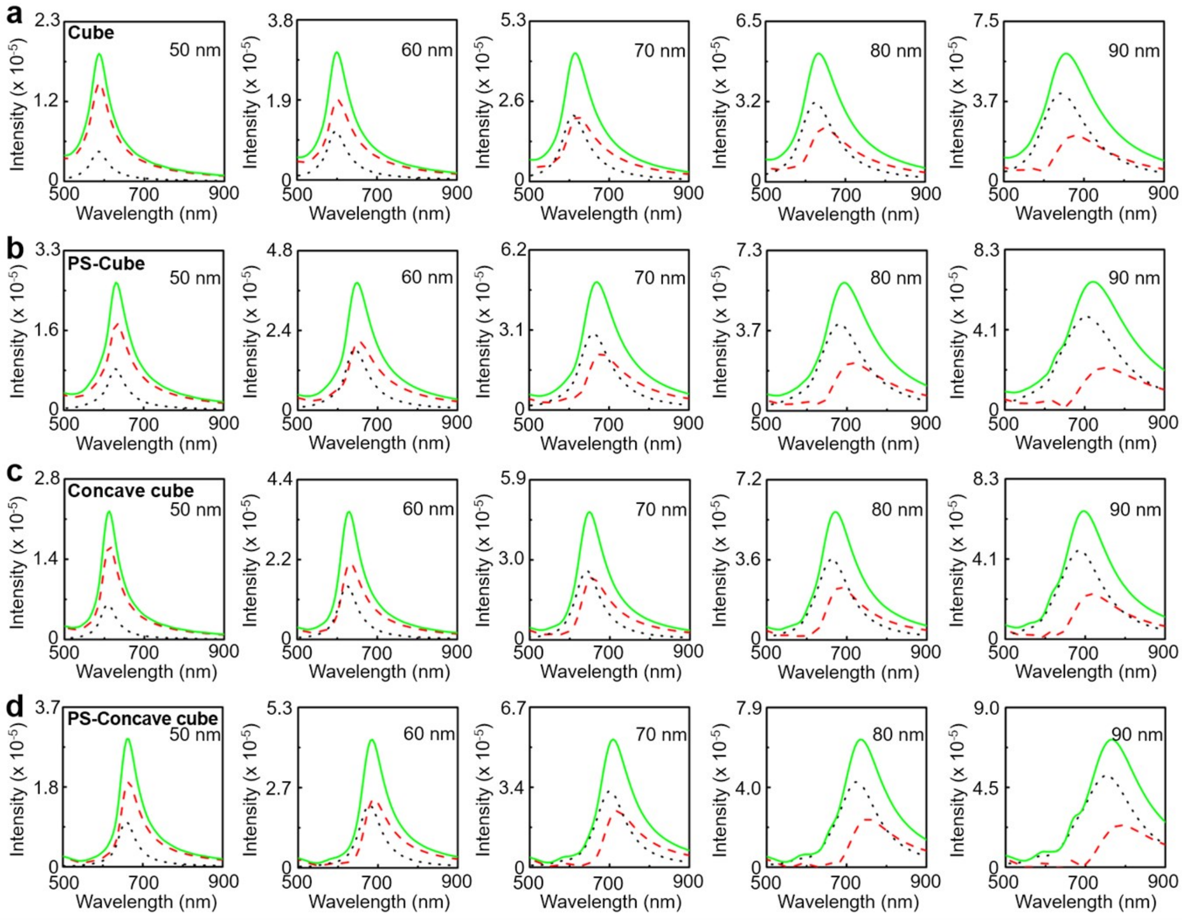

Fig. S10 Simulated absorption (red dotted line), scattering (black dotted line) and extinction (green solid line) spectra of (a) gold nanocubes, (b) PS-grafted gold nanocubes, (c) gold concave nanocubes and (d) PS-grafted gold concave nanocubes with different edge lengths (from left to right: 50 nm, 60 nm, 70 nm, 80 nm and 90 nm). The PS shell thickness was set as 20 nm.

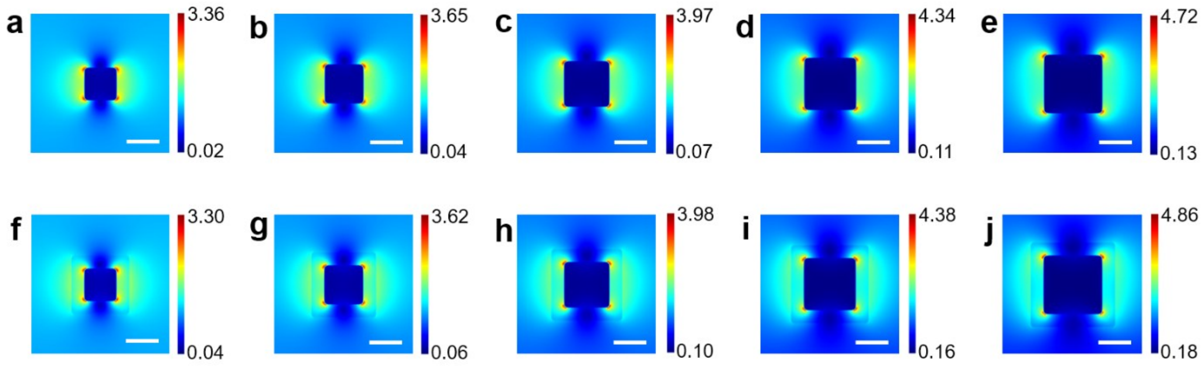

Fig. S11 (a-e) Two-dimensional electric field map of gold nanocubes with different edge lengths ((a): 50 nm, (b): 60 nm, (c): 70 nm, (d): 80 nm and (e): 90 nm in edge length). (f-j) Two-dimensional electric field map of PS-grafted gold nanocubes with different edge lengths ((f): 50 nm, (g): 60 nm, (h): 70 nm, (i): 80 nm and (j): 90 nm in edge length). The electric field maps of gold nanocubes before the PS grafting were simulated in water, and those of PS-grafted gold nanocubes (PS shell thickness = 20 nm) were simulated in DMF. Scale bars: 50 nm.

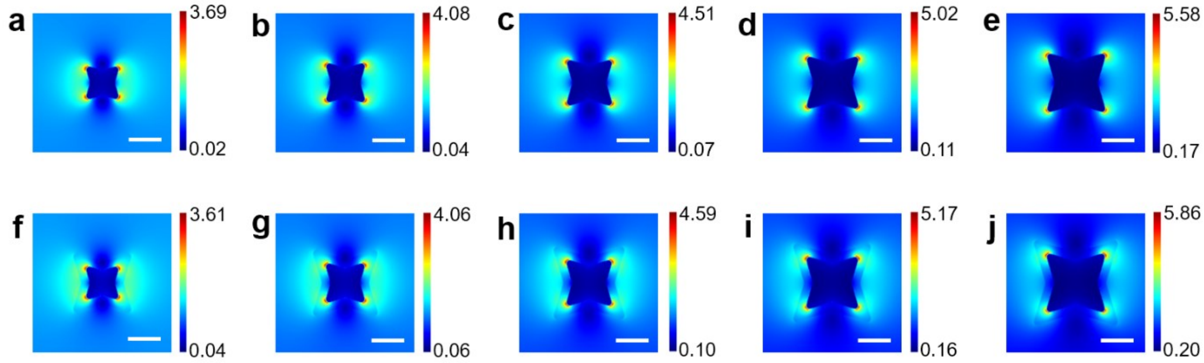

Fig. S12 (a-e) Two-dimensional electric field map of gold concave nanocubes with different edge lengths ((a): 50 nm, (b): 60 nm, (c): 70 nm, (d): 80 nm and (e): 90 nm in edge length). (f-j) Two-dimensional electric field map of PS-grafted gold concave nanocubes with different edge lengths ((f): 50 nm, (g): 60 nm, (h): 70 nm, (i): 80 nm and (j): 90 nm in edge length). The electric field maps of gold concave nanocubes before the PS grafting were simulated in water, and those of PS-grafted gold concave nanocubes (PS shell thickness = 20 nm) were simulated in DMF. Scale bars: 50 nm.

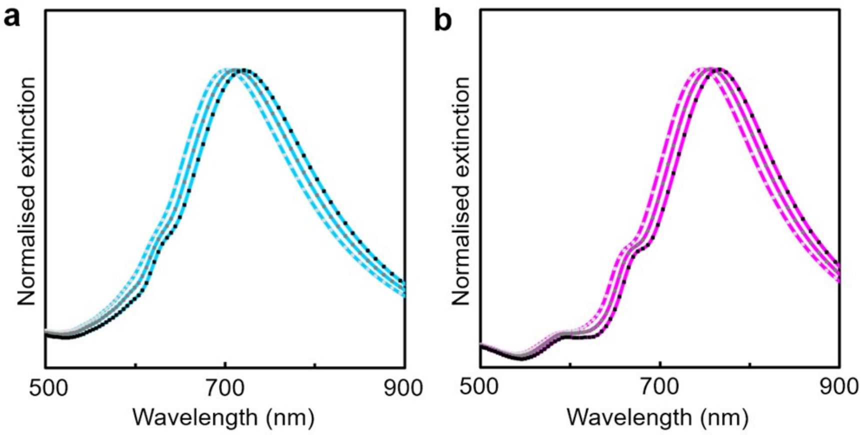

Fig. S13 (a) Simulated UV-visible spectra of PS-grafted gold nanocubes with different PS shell thicknesses (from left to right: 6 nm, 12 nm and 20 nm in PS shell thickness). (b) Simulated UV-visible spectra of PS-grafted gold concave nanocubes with different PS shell thicknesses (from left to right: 6 nm, 12 nm and 20 nm in PS shell thickness). The core edge length was set as 90 nm, and the spectra were simulated in DMF.

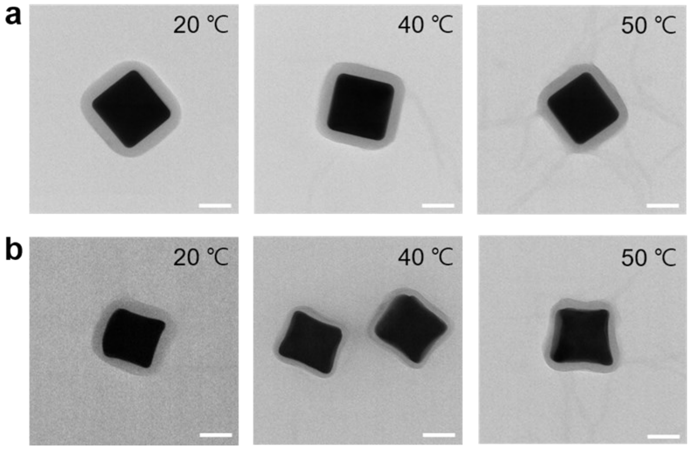

Fig. S14 (a) TEM images of PS-grafted gold nanocubes treated at different temperatures (from left to right: 20 ℃, 40 ℃ and 50 ℃). (b) TEM images of PS-grafted gold concave nanocubes treated at different temperatures (from left to right: 20 ℃, 40 ℃ and 50 ℃). Scale bars: 50 nm.

Sample	Core edge length (nm)	Shell thickness (nm)	$Volume_{Core}$ (nm^3)	$Volume_{Shell}$ (nm^3)	$Volume_{Shell}$ / $Volume_{Core}$
**PS-Cube**	51.5	18.3	136569	548719	4.02
	60.0	19.7	215895	783813	3.63
	68.1	19.4	316139	924639	2.92
	78.3	19.3	479601	1119644	2.33
	87.9	19.8	678221	1379523	2.03
**PS-Concave cube**	49.6	19.4	84717	392722	4.64
	59.8	19.6	148468	514353	3.46
	68.6	19.7	224130	648831	2.89
	78.3	19.0	333283	758829	2.28
	87.5	18.2	465106	863949	1.86

Table S1 Dimensional analysis of PS-grafted gold polyhedra.

References

1 M. N. O'Brien, M. R. Jones, K. A. Brown and C. A. Mirkin, *J. Am. Chem. Soc.*, 2014, **136**, 7603–7606.

2 J. Kim, X. Song, A. Kim, B. Luo, J. W. Smith, Z. Ou, Z. Wu and Q. Chen, *Macromol. Rapid Commun.*, 2018, **39**, 1800101.

3 A. Klinkova, H. Thérien-Aubin, A. Ahmed, D. Nykypanchuk, R. M. Choueiri, B. Gagnon, A. Muntyanu, O. Gang, G. C. Walker and E. Kumacheva, *Nano Lett.*, 2014, **14**, 6314–6321.

4 P. B. Johnson and R. W. Christy, *Phys. Rev. B*, 1972, **6**, 4370–4379.

5 N. G. Sultanova, S. N. Kasarova and I. D. Nikolov, *Opt. Quantum Electron.*, 2013, **45**, 221–232.
